# *OsSLC1* Encodes a Pentatricopeptide Repeat Protein Essential for Early Chloroplast Development and Seedling Survival

**DOI:** 10.1186/s12284-020-00385-5

**Published:** 2020-04-15

**Authors:** Jun Lv, Lianguang Shang, Yun Chen, Yao Han, Xiaoyan Yang, Shuzhang Xie, Wenqin Bai, Mingyu Hu, Hong Wu, Kairong Lei, Ya’nan Yang, Shengzhen Ge, Hai Phuong Trinh, Yi Zhang, Longbiao Guo, Zhongwei Wang

**Affiliations:** 1 College of Agronomy and Biotechnology, Southwest University, Chongqing, 400715 China; 2 Shenzhen Branch, Guangdong Laboratory for Lingnan Modern Agriculture, Genome Analysis Laboratory of the Ministry of Agriculture, Agricultural Genomics Institute at Shenzhen, Chinese Academy of Agricultural Sciences, Shenzhen, 518124 China; 3 State Key Laboratory for Conservation and Utilization of Bio-resources in Yunnan, Research Center for Perennial Rice Engineering and Technology in Yunnan, School of Agriculture, Yunnan University, Kunming, 650500 China; 4 Chongqing Key Laboratory of Adversity Agriculture Research, Biotechnology Research Center, Chongqing Academy of Agricultural Sciences, Chongqing, 401329 China; 5 State Key Laboratory of Rice Biology, China National Rice Research Institute, Zhejiang, 310006 China

**Keywords:** *Oryza sativa*, Chlorosis phenotype, PPR protein, Intron splicing, Chloroplast development

## Abstract

**Background:**

The large family of pentatricopeptide repeat (PPR) proteins is widely distributed among land plants. Such proteins play vital roles in intron splicing, RNA editing, RNA processing, RNA stability and RNA translation. However, only a small number of PPR genes have been identified in rice.

**Results:**

In this study, we raised a mutant from tissue-culture-derived plants of *Oryza sativa subsp. japonica* ‘Zhonghua 11’, which exhibited a lethal chlorosis phenotype from germination to the third-leaf stage. The mutant was designated *seedling-lethal chlorosis 1* (*slc1*). The *slc1* mutant leaves showed extremely low contents of photosynthetic pigments and abnormal chloroplast development, and were severely defective in photosynthesis. Map-based cloning of *OsSLC1* revealed that a single base (G) deletion was detected in the first exon of *Os06g0710800* in the *slc1* mutant, which caused a premature stop codon. Knockout and complementation experiments further confirmed that *OsSLC1* is responsible for the seedling-lethal chlorosis phenotype in the *slc1* mutant. *OsSLC1* was preferentially expressed in green leaves, and encoded a chloroplast-localized PPR protein harboring 12 PPR motifs. Loss-of-function of *OsSLC1* affected the intron splicing of multiple group II introns, and especially precluded the intron splicing of *rps16*, and resulted in significant increase in the transcript levels of 3 chloroplast ribosomal RNAs and 16 chloroplast development-related and photosynthesis-related genes, and in significant reduction in the transcript levels of 1 chloroplast ribosomal RNAs and 2 chloroplast development-related and photosynthesis-related genes.

**Conclusion:**

We characterized a novel chloroplast-localized PPR protein, OsSLC1, which plays a vital role in the intron splicing of multiple group II introns, especially the *rps16* intron, and is essential for early chloroplast development and seedling survival in rice.

## Background

The chloroplast is a vital photosynthetic organelle for plant growth and development. The plant chloroplast is predicted to contain approximately 2500–3000 proteins, of which less than 10% are encoded by the chloroplast genome, the majority being encoded by the nuclear genome (Race et al. 1999; Armbruster et al. 2011). Introns are prevalent in plant chloroplast genomes, and intron splicing is an essential step prior to RNA translation.

The rice chloroplast genome is about 135 kb in size, consisting of approximately 128 genes, and 17 *cis*-spliced introns and one *trans*-spliced intron are distributed across 15 genes (Hiratsuka et al. 1989; Wu and Ge 2016). Among these introns, one in the *trnL* gene belongs to group I, with the remaining introns belonging to group II. However, the ability of these introns to perform self-splicing in vivo has been lost, and thus additional nuclear- and/or plastid-encoded splicing cofactors are required to accomplish intron splicing (Bonen and Vogel 2001; de Longevialle et al. 2010). Such cofactors include the plastid maturase matK, chloroplast RNA splicing and ribosome maturation proteins, pentatricopeptide repeat (PPR) proteins, RNA helicases, mitochondrial transcription termination factors, a RNase III domain-containing protein, WHAT IS THIS FACTOR 1 (WTF1) and a WHY and UMP kinase (de Longevialle et al. 2010; Quesada 2016; Nawaz and Kang 2017; Schmid et al. 2019). Of these cofactors, PPR proteins not only play vital roles in intron splicing, but also in RNA editing, RNA processing, RNA stability and RNA translation (Barkan and Small 2014).

A large family of PPR proteins is widely distributed among land plants. Approximately 477 PPR proteins are known in the rice nuclear genome (Lurin et al. 2004; Barkan and Small 2014). Generally, PPR proteins are divided into P (only containing PPR motifs) and PLS (containing PPR, PPR-like L (for long) and S (for short) motifs) subgroups, and on the basis of C-terminal motifs, the PLS subgroup is further subdivided into PLS, E/E+ (containing E/E+ motifs) and DYW (containing DYW motifs) subgroups (Lurin et al. 2004). To date, only a small number of PPR genes, comprising five restorer-of-fertility genes and approximately 25 additional PPR genes, have been identified in rice.

Among the identified PPR proteins, 11 are reported to be involved in intron splicing. OsOTP51 is involved in the intron splicing of *atpF*, especially *ycf-2*, in chloroplasts (Ye et al. 2012). The mitochondrion-localized OsMPR25 is involved in the intron splicing of *atpF* in chloroplasts (Toda et al. 2012; Yap et al. 2015). OsPPR4 (Asano et al. 2013) and OsWSL4 (Wang et al. 2017a) are both predominantly involved in the intron splicing of *atpF*, *ndhA*, *rpl2* and *rps12* in chloroplasts. The chloroplast-localized OsWSL is involved in the intron splicing of *rpl2* in chloroplasts (Tan et al. 2014). The chloroplast-localized OsPPR6 is involved in the intron splicing of *ycf3* in chloroplasts (Tang et al. 2017). The chloroplast-localized OsSLA4 is mainly involved in the intron splicing of *atpF*, *ndhA*, *petB*, *rpl2*, *rpl16*, *rps12* and *trnG* in chloroplasts (Wang et al. 2018). The chloroplast-localized OsWSL5 is predominantly involved in the intron splicing of *rpl2* and *rps12* in chloroplasts (Liu et al. 2018). The chloroplast-localized OsPGL12 is mainly involved in the intron splicing of *ndhA* in chloroplasts (Chen et al. 2019). The mitochondrion-localized OsFLO10 is mainly involved in the *trans*-splicing of the *nad1* intron 1 in mitochondria (Wu et al. 2019), and the nucleus-localized OsNPPR1 was reported to be involved in the intron splicing of a small number of nuclear-localized genes, many of which are mitochondrion-localized (Hao et al. 2019). The chloroplast-localized and/or mitochondrion-localized PPR proteins, such as OsPPR1 (Gothandam et al. 2005), OsOTP51, OsPPR4, OsWSL, OsALS3 (Lin et al. 2015a), OspTAC2 (Wang et al. 2016a), OsPPR6 (Tang et al. 2017), OsSLA4, OsWSL5 and OsPGL12, are essential for early chloroplast development in rice at the seedling stage.

In the present study, we characterized a novel PPR gene, *OsSLC1*, from a rice *seedling-lethal chlorosis 1* (*slc1*) mutant. *OsSLC1* encodes a chloroplast-localized P subgroup PPR protein harboring 12 PPR motifs. Analysis of loss-of-function of the *slc1* mutant revealed that OsSLC1 plays a vital role in the intron splicing of multiple group II introns, especially the *rps16* intron, and is essential for early chloroplast development and seedling survival in rice.

## Results

### The *slc1* Mutant Exhibits a Seedling-Lethal Chlorosis Phenotype

**Fig. 1:**
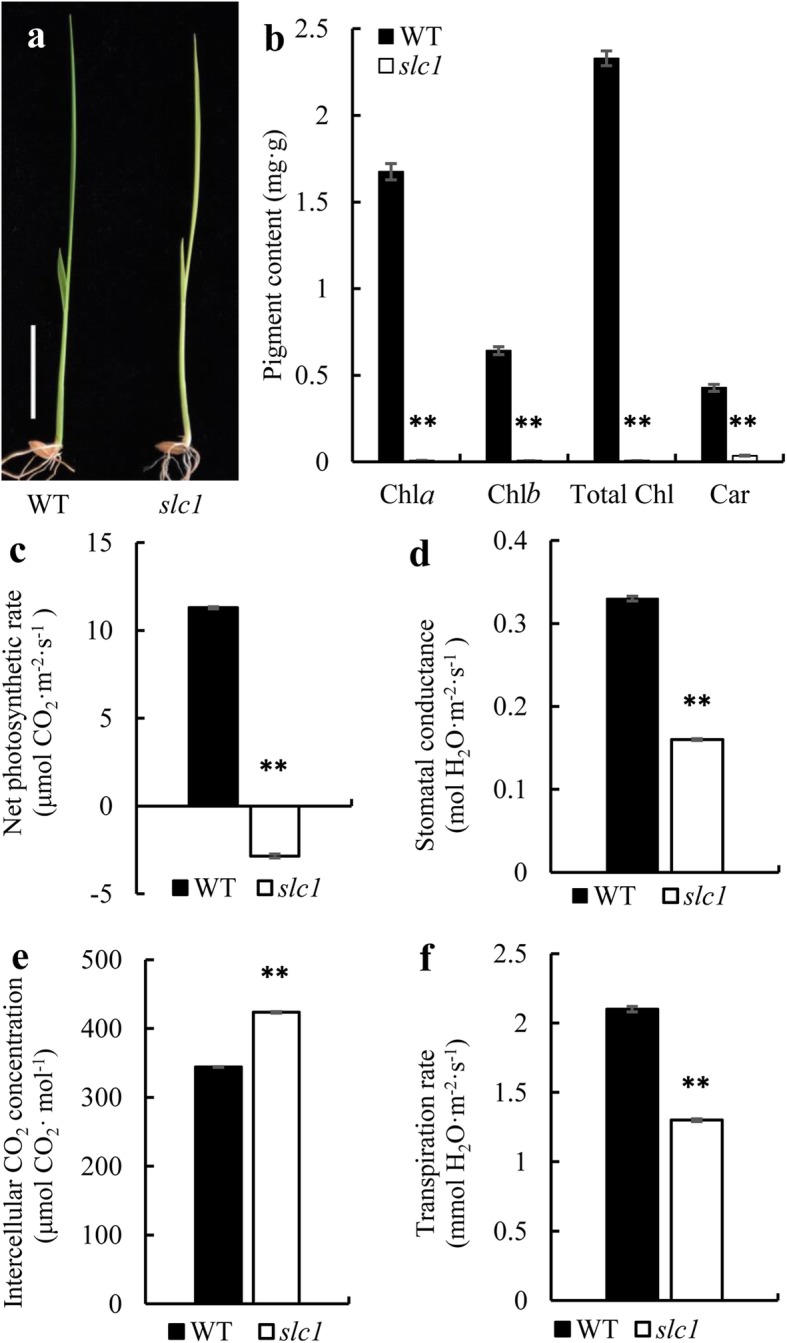
Phenotypic characteristics of the *slc1* mutant and measurement of leaf photosynthetic parameters in the wild type (WT) and *slc1* plants at the seedling stage. **a** Phenotypes of the wild type and *slc1* plants at the second-leaf stage. Scale bar, 2 cm. **b** Leaf photosynthetic pigment contents of the WT and *slc1* plants at the third-leaf stage. Chl, chlorophyll; Car, carotenoid. **c** The net photosynthetic rate. **d** The stomatal conductance. **e** The intercellular CO_2_ concentration. **f** The transpiration rate. Three biological replicates were performed. Error bars are SDs. The asterisks indicate statistical significance between the wild type and *slc1* mutant, as determined by Student’s t-test (** *P* < 0.01)

The *slc1* mutant was obtained from tissue-culture-derived plants of rice *Oryza sativa subsp. japonica* ‘Zhonghua 11’, which exhibited a chlorosis phenotype from germination to the third-leaf stage, and seedlings gradually died off around 1 month after germination. The *slc1* mutant at the second-leaf stage is shown in Fig. 1a. The chlorophyll *a*, chlorophyll *b* and carotenoid contents were extremely low in the *slc1* mutant compared with the wild type (Fig. 1b).

### The *slc1* Mutant Shows Severe Defects in Photosynthesis and Chloroplast Development

**Fig. 2:**
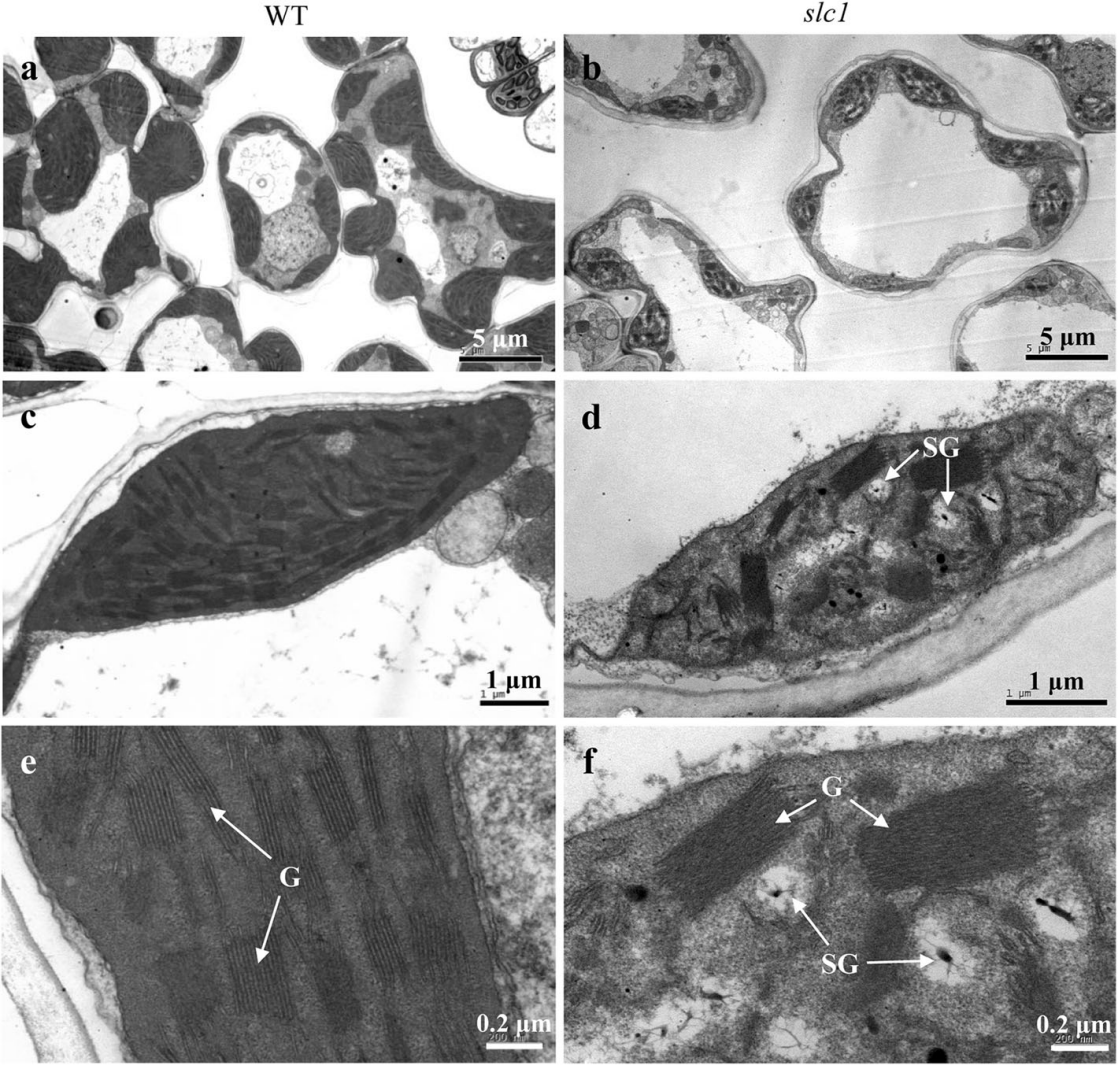
Ultrastructure of chloroplasts in the wild type (WT) (**a**, **c**, **e**) and *slc1* mutant leaves (**b**, **d**, **f**) at the third-leaf stage. G, grana; SG, starch grain. Scale bars are shown bottom right

To investigate whether leaf photosynthesis was affected in the *slc1* mutant, photosynthetic parameters were measured. The net photosynthetic rate, stomatal conductance and transpiration rate were significantly decreased (Fig. 1c, d, f), and the intercellular CO_2_ concentration was significantly increased in the *slc1* mutant compared with the wild type (Fig. 2e). These results indicate that the *slc1* mutant is severely defective in photosynthesis.

To investigate whether chloroplast development was affected in the *slc1* mutant leaves, the chloroplast ultrastructure was observed. The leaf mesophyll cells contained large vesicles, and the chloroplasts exhibited normal shapes but accumulated some starch grains, and contained indistinct thylakoids and a small number of grana stacks in the *slc1* mutant (Fig. 2b, d, f); in contrast, the leaf mesophyll cells contained normal-sized vesicles, and the chloroplasts contained distinct thylakoids and a large number of regular and dense grana stacks in the wild type (Fig. 2a, c, e). These results indicate that chloroplast development is severely impaired in the *slc1* mutant.

### Map-Based Cloning of *OsSLC1*

All heterozygous F_1_ plants showed a normal green phenotype and segregation occurred in F_2_ plants. The segregation ratio of 3:1 (green:chlorotic = 338:126; χ^2^ = 1.14 < χ^2^_0.05_ = 3.84) indicates that a single recessive locus results in the seedling-lethal chlorosis phenotype in the *slc1* mutant. The locus was designated *OsSLC1*.

**Fig. 3:**
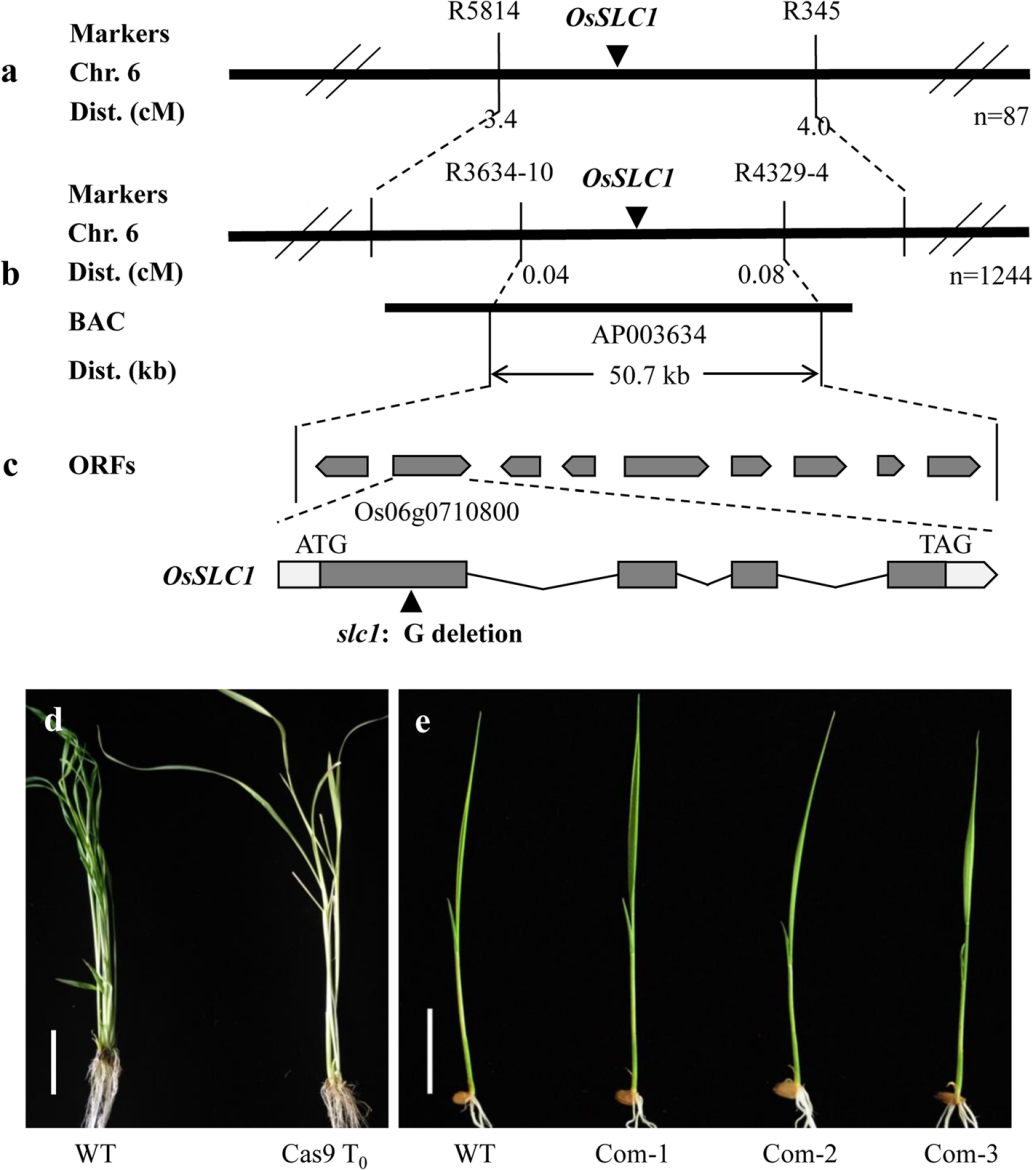
Map-based cloning of *OsSLC1*. **a** The *OsSLC1* locus was mapped to chromosome 6 (Chr. 6). **b** The *OsSLC1* locus was fine-mapped to a 50.7 kb interval on the BAC clone AP003634. **c** Open reading frame of the candidate gene *Os06g0710800* in the mapped region; the mutation site is indicated by a black upright triangle. **d** T_0_ mutant generated using the CRISPR/Cas9 system. **e** Independent T_1_ complementation plants. WT, wild type. Scale bars, 2 cm

Using 87 chlorotic mutants, the *OsSLC1* locus was initially mapped to chromosome 6 between markers R5814 and R345 (Fig. 3a). To constrain the interval containing the *OsSLC1* locus, an additional F_2_ population with 1244 chlorotic mutants was used for fine mapping. The *OsSLC1* locus was mapped to a 50.7 kb interval between markers R3634–10 and R4329–4 on the BAC clone AP003634 (Fig. 3b). This region contains a total of nine putative open reading frames (Fig. 3c). Sequencing analysis revealed that a single-base (G) deletion was detected in the first exon of *Os06g0710800* in the *slc1* mutant (Fig. 3c), which caused a premature stop codon. *Os06g0710800* was predicted to contain four exons with a coding sequence of 1479 bp. Accordingly, *Os06g0710800* was selected as the candidate gene for *OsSLC1*.

To investigate whether the loss-of-function of *Os06g0710800* results in the chlorosis phenotype in the *slc1* mutant, the non-mutated *Os06g0710800* gene of the wild type ‘Zhonghua 11’ was knocked out using a CRISPR/Cas9 system. Nine homologous T_0_ transgenic plants displayed the same seedling-lethal chlorosis phenotype. To further confirm the function of *Os06g0710800*, the OsSLC1-EGFP fusion cassette was transformed into the *slc1* calli. A total of 19 of 21 T_0_ transgenic plants rescued the normal wild-type phenotype. The T_0_ knock-out line and independent T_1_ complementation plants are illustrated in Fig. 3d and e, respectively. These results indicate that *Os06g0710800* is the candidate *OsSLC1* gene, and is responsible for the seedling-lethal chlorosis phenotype in the *slc1* mutant.

### *OsSLC1* Is Preferentially Expressed in Green Leaves and Encodes a Chloroplast-Localized Protein

**Fig. 4:**
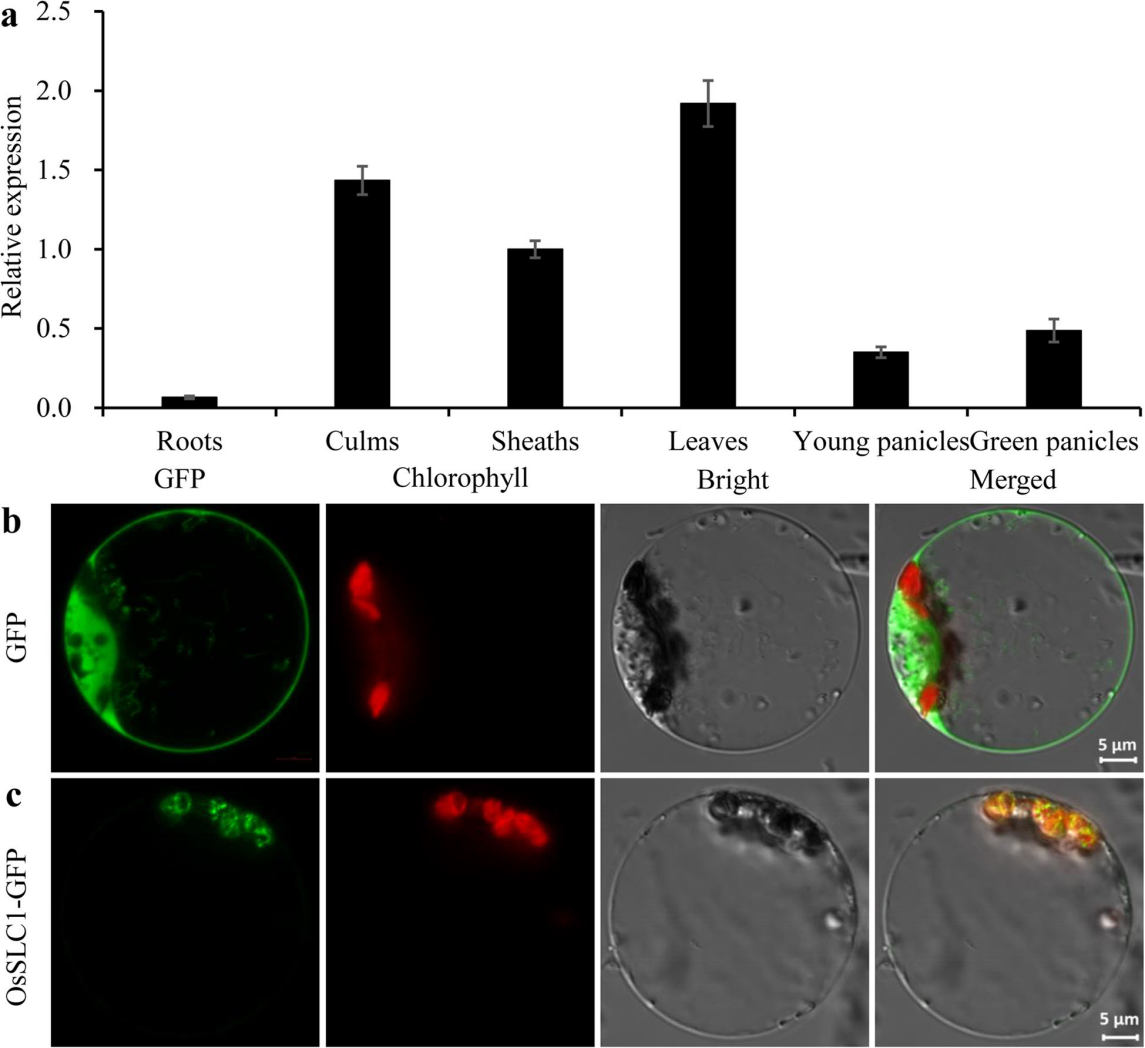
Expression profile analysis of *OsSLC1* in the wild type plant and subcellular localization of OsSLC1 in rice protoplasts. **a** Expression analysis of *OsSLC1* in a variety of wild type tissues. RNAs were extracted from roots, culms, sheaths, leaves, young panicles (white) and green panicles at the heading stage. **b** Observation of free green fluorescent protein (GFP) signals in rice protoplasts. **c** Observation of OsSLC1-GFP fluorescent signals in rice protoplasts. Scale bars, 5 μm

To investigate the expression pattern of *OsSLC1* in the wild type, quantitative real-time PCR was performed to analyze the relative expression level of *OsSLC1* in a variety of tissues. *OsSLC1* was predominantly expressed in the culm, sheath and green leaf, but was relatively highly expressed in green leaves (Fig. 4a).

The majority of PPR proteins contain a chloroplast- or mitochondrion-targeting peptide. To determine whether OsSLC1 was localized to the chloroplasts or mitochondria, the pOsSLC1-GFP vector was introduced into rice protoplasts. The green fluorescent signals from the OsSLC1-GFP fusion proteins overlapped with the chloroplast autofluorescences (Fig. 4b). These results indicate that OsSLC1 is localized to the chloroplasts.

### OsSLC1 Is a Member of the P Subgroup of PPR Proteins

**Fig. 5:**
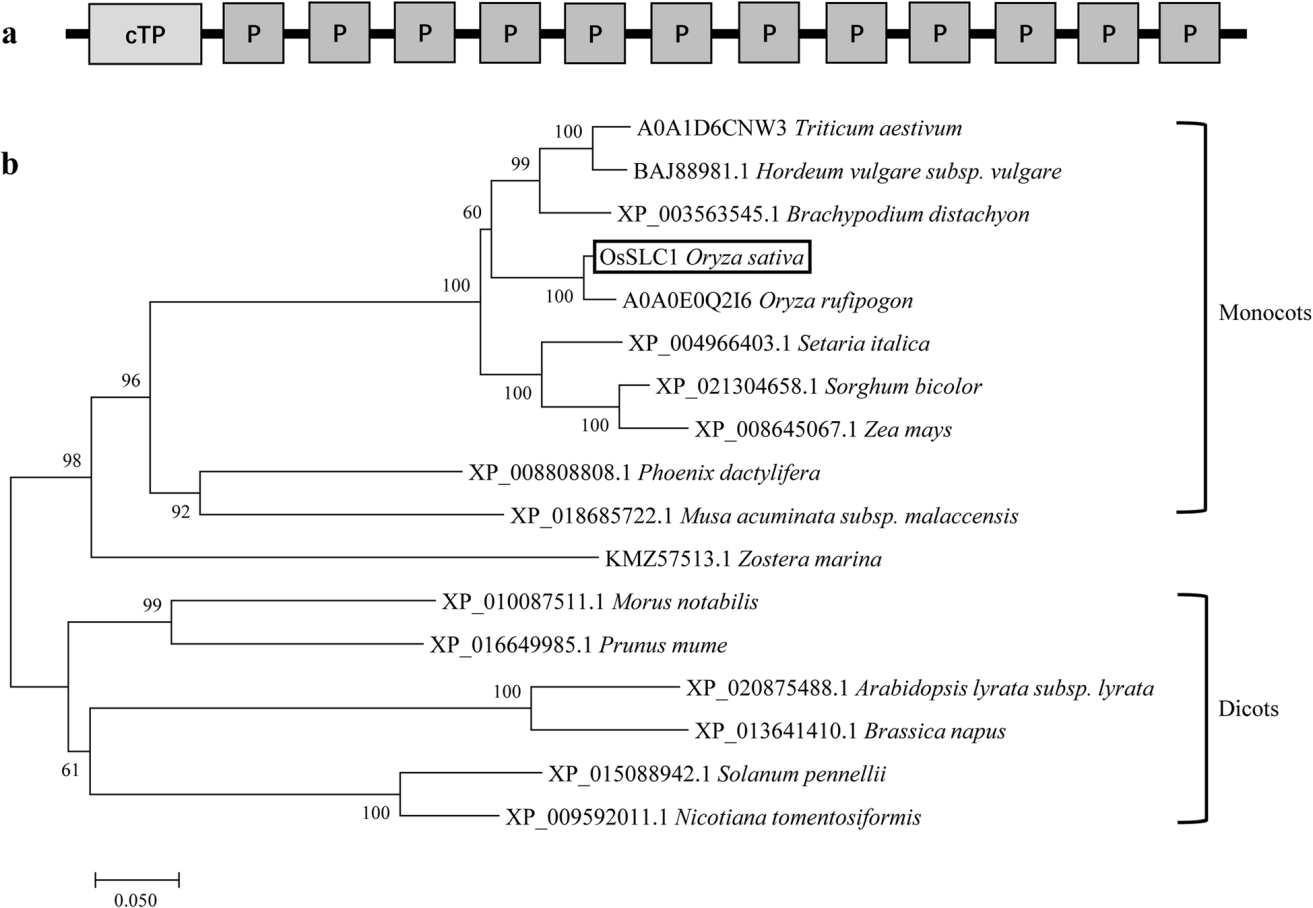
Structure and phylogenetic analysis of OsSLC1. **a** Predicted structure of OsSLC1. OsSLC1 contains a chloroplast transit peptide (cTP) and 12 PPR motifs (P). **b** Phylogenetic tree of OsSLC1 and its homologs. The protein sequences of A0A1D6CNW3 (*Triticum aestivum*) and A0A0E0Q2I6 (*Oryza rufipogon*) were downloaded from the UniProt database; all other sequences were downloaded from the National Center for Biotechnology Information database

OsSLC1 was predicted to be a PPR protein composed of 492 amino acids. Protein sequence analysis showed that OsSLC1 contained a chloroplast transit peptide and 12 PPR motifs, and thus belonged to the P subgroup of the PPR family (Fig. 5a). Phylogenetic analysis showed that a class of unreported proteins, which were homologous to OsSLC1, were clustered into monocotyledon and dicotyledon groups (Fig. 5b). These results indicate that a novel class of unknown function of proteins homologous to OsSLC1 are widely distributed among angiosperms.

### The *slc1* Mutant Is Defective in the Intron Splicing of Multiple Group II Introns, Especially the *rps16* Intron

**Fig. 6:**
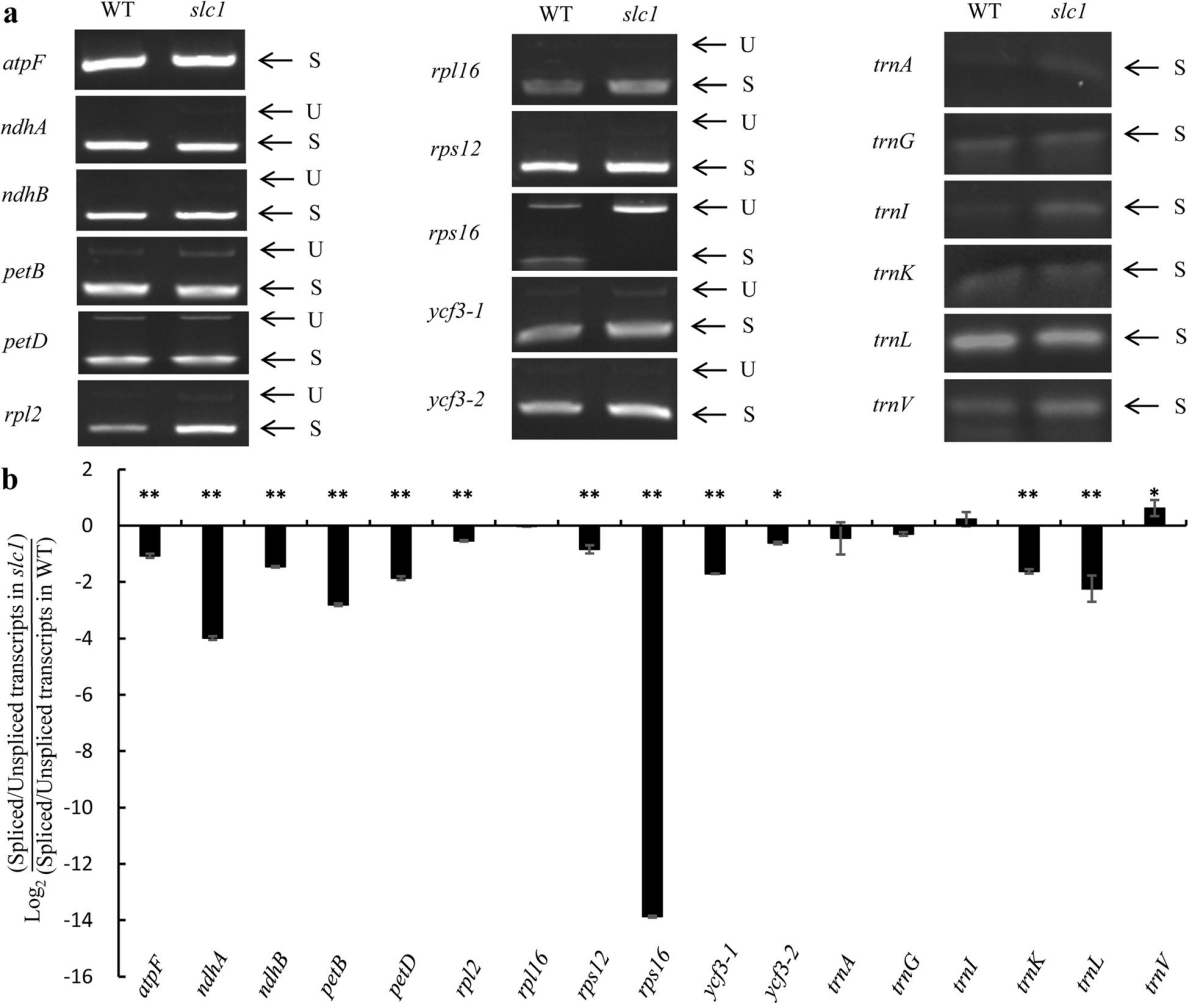
Splicing analysis of chloroplast intron-containing transcripts in the wild type and *slc1* mutant leaves at the third-leaf stage. **a** RT-PCR analysis of 18 intron-containing genes in the wild type and *slc1* mutant leaves. U, Unspliced transcripts; S, Spliced transcripts. **b** Splicing efficiency analysis of chloroplast intron-containing transcripts in the wild type and *slc1* mutant leaves. *psbA* was used as internal control, and relative transcript levels were analyzed using the 2^-ΔΔCT^ method (Livak and Schmittgen 2001). Three biological replicates were performed. Values indicating the log_2_ ratios of spliced to unspliced transcripts in the *slc1* mutant compared to the corresponding values in the wild type. Error bars are SDs. The asterisks indicate statistical significance between the wild type and *slc1* mutant, as determined by Student’s t-test (* *P* < 0.05, ** *P* < 0.01)

The P subgroup of PPR proteins are generally involved in intron splicing, RNA processing, RNA stability and RNA translation (Barkan and Small 2014; Manna 2015). Firstly, RT-PCR and quantitative real-time PCR were used to analyze the intron splicing of all 18 intron-containing chloroplast genes. The RT-PCR results showed that the intron splicing of *rps16* was precluded, whereas other introns were naturally spliced in the *slc1* mutant compared with the wild type (Fig. 6a). The quantitative real-time PCR results showed that the transcript levels of the spliced *rps16 w*as severely reduced, and those of the spliced *ndhA* and *petB* were also variously reduced, whereas those of the spliced *atpF*, *ndhB*, *petD*, *rpl2*, *rpl16*, *rps12*, *ycf3–1*, *ycf3–2*, *trnA*, *trnG*, *trnI*, *trnK* and *trnV* were distinctly increased, and those of the other spliced genes analyzed did not change that much in the *slc1* mutant compared with the wild type (Supplementary Figure S1). Further, quantitative real-time PCR was used to analyze the intron splicing efficiency of 18 intron-containing genes. The intron splicing efficiency of *rps16* was extremely reduced, and those of *atpF*, *ndhA*, *ndhB*, *petB*, *petD*, *rpl2*, *rps12*, *ycf3–1*, *ycf3–2*, *trnA*, *trnK* and *trnL* were variously reduced (Fig. 6b), but most of their spliced mature transcripts were variously increased or not notably changed in the *slc1* mutant compared with the wild type (Fig. 6a, Supplementary Figure S1). In addition, we examined 26 chloroplast RNA editing sites in the wild type and *slc1* mutant. The majority of the editing sites were completely edited except for a few minor changes in the *slc1* mutant compared with the wild type (Supplementary Table S1). Taken together, these results indicate that *OsSLC1* plays a vital role in the intron splicing of multiple group II introns, especially the *rps16* intron, rather than RNA editing, in rice.

### Transcript Levels of Chloroplast Ribosomal RNAs, and Chloroplast Development-Related and Photosynthesis-Related Genes Are Altered in the *slc1* Mutant

**Fig. 7:**
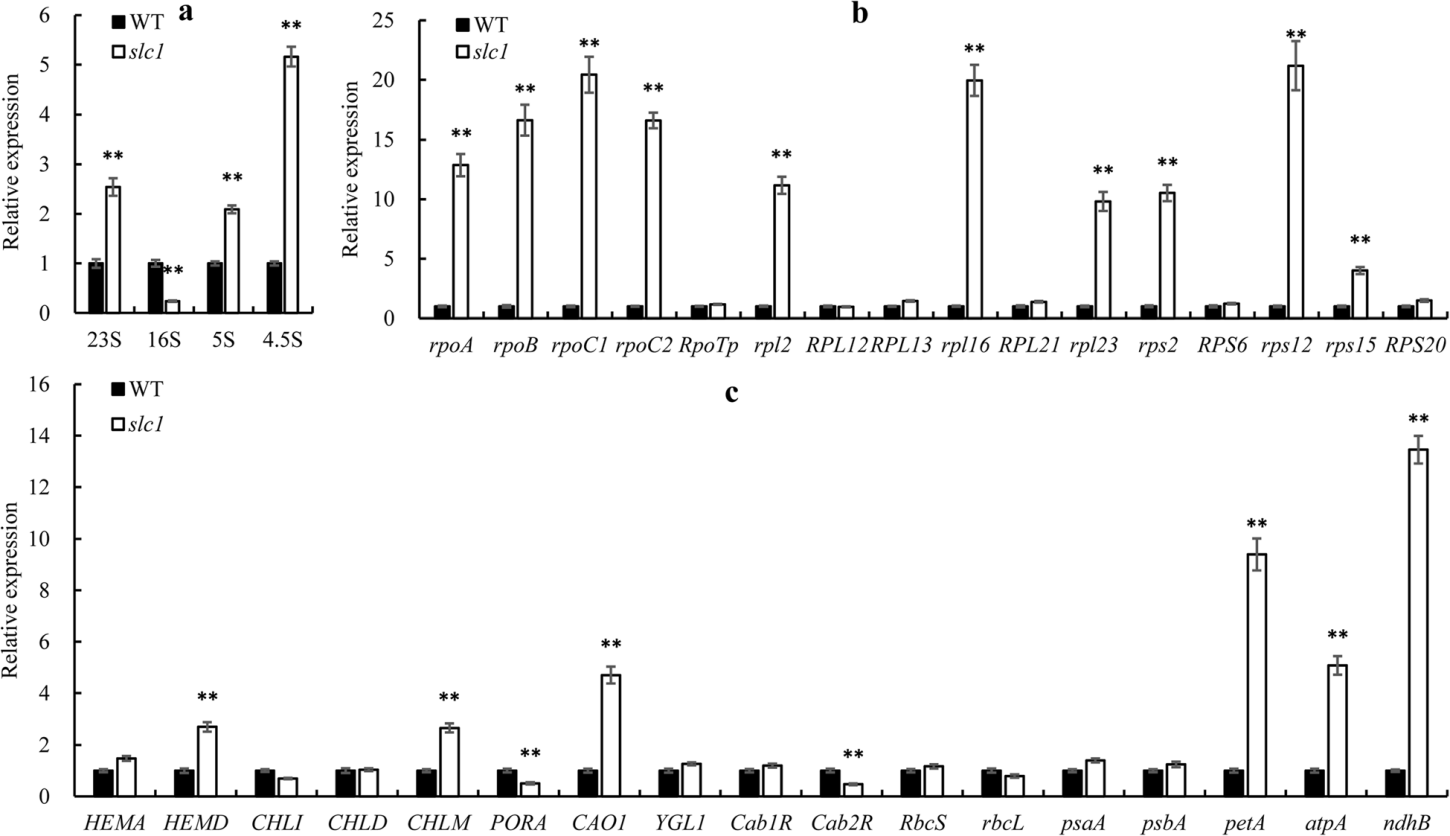
Quantitative real-time PCR analysis of genes associated with chloroplast development and photosynthesis in the wild type and *slc1* mutant leaves at the third-leaf stage. **a** Transcript analysis of chloroplast 23S, 16S, 5S and 4.5S rRNAs. **b** Transcript analysis of plastid-encoded and nucleus-encoded chloroplast development-related genes. **c** Transcript analysis of chlorophyll synthesis-related and photosynthesis-related genes. *OsActin1* and *OsUbiquitin* were used as double internal controls, and relative transcript levels were analyzed using the 2^-ΔΔCT^ method (Livak and Schmittgen 2001). Three biological replicates were performed. Error bars are SDs. The asterisks indicate statistical significance between the wild type and *slc1* mutant, as determined by Student’s t-test (** *P* < 0.01)

To investigate whether the expression levels of chloroplast and nuclear genes were affected in the *slc1* mutant, chloroplast ribosomal RNAs and chloroplast development-related and photosynthesis-related genes were detected in the wild type and *slc1* mutant. The transcript levels of 23S, 5S and 4.5S rRNAs were significantly increased, whereas those of 16S rRNA was significantly reduced in the *slc1* mutant (Fig. 7a). The transcript levels of the plastid-encoded RNA polymerase (PEP) genes *rpoA*, *rpoB*, *rpoC1* and *rpoC2* were significantly increased, whereas those of the nuclear-encoded RNA polymerase gene *RpoTp* was hardly changed in the *slc1* mutant (Fig. 7b). The transcript levels of the nuclear-encoded chloroplast large 50S and small 30S subunits genes *RPL12*, *RPL13*, *RPL21*, *RPS6* and *RPS20* were not notably changed, whereas those of the plastid-encoded large 50S and small 30S subunits genes *rpl2*, *rpl16*, *rpl23*, *rps2*, *rps12* and *rps15* were significantly increased in the *slc1* mutant (Fig. 7b). The transcript levels of the chloroplast development-related and photosynthesis-related genes *PORA* and *Cab2R* were significantly reduced, whereas those of *HEMD*, *CHLM*, *CAO1*, *petA*, *atpA* and *ndhB* were significantly increased, and those of the other genes analyzed did not change that much in the *slc1* mutant (Fig. 7c). These results indicate that *OsSLC1* plays an important role in transcript expression of chloroplast development-related and photosynthesis-related genes in rice.

## Discussion

### PPR Proteins Are Essential for Normal Plant Growth and Development

Although the large PPR family is widely distributed among plants, only a small number of PPR proteins have been characterized. The majority of PPR proteins contain chloroplast- or mitochondrion-targeting peptides (Lurin et al. 2004), and some contain nucleus-targeting peptides (Ding et al. 2006; Hao et al. 2019). PPR proteins commonly participate in RNA metabolism in organelles or the nucleus, and are essential for normal plant growth and development.

Mutations of PPR genes in rice typically cause a variety of abnormal phenotypes, such as seedling lethality, abnormal seedling growth, delayed seed germination, retarded growth, dwarfism, defective seed development, embryo lethality and sterility. For example, mutations of chloroplast-localized PPR genes generally cause seedling-lethal albino or chlorosis phenotypes under natural conditions, as is the case for *OsPPR1* (Gothandam et al. 2005), *OsOTP51* (Ye et al. 2012), *OsPPR4* (Asano et al. 2013), *OsASL3* (Lin et al. 2015a), *OspTAC2* (Wang et al. 2016a), *OsPPR6* (Tang et al. 2017) and *OsSLA4* (Wang et al. 2018). Loss-of-function of *OsSLC1* also causes a seedling-lethal chlorosis phenotype in the *slc1* mutant. Mutations of mitochondrion-localized PPR genes typically cause retarded growth, dwarfism, defective seed development, as is the case for *OsOGR1* (Kim et al. 2009), *OsEMP5* (Liu et al. 2013), *OsSMK1* (Li et al. 2014) and *OsPPS1* (Xiao et al. 2018).

### OsSLC1 Is Essential for Early Chloroplast Development and Seedling Survival

The chloroplast possesses its own protein translation system. The bacterial-type chloroplast 70S ribosome, consisting of 30S small and 50S large subunits, is essential for protein translation in chloroplasts (Harris et al. 1994). The 30S ribosomal subunit, composed of 24 proteins, recruits the 16S rRNA to initiate the translation of mRNA, whereas the large 50S ribosomal subunit, composed of 33 proteins, recruits the 23S, 5S and 4.5S rRNAs to activate the initiation of translation of mRNA (Yamaguchi et al. 2000; Yamaguchi and Subramanian 2000; Tiller and Bock 2014; Bieri et al. 2017). The 16S rRNA plays a vital role in mRNA binding and stabilization of codon-anticodon interaction (Bieri et al. 2017). Previous studies indicate that loss-of-function of genes that encode the 30S small or 50S large subunits always causes abnormal chloroplast development and seedling growth in rice.

Mutation of *OsASL1*, which encodes the 30S small subunit protein RPS20, causes impaired chloroplast development and a seedling-lethal albino phenotype (Gong et al. 2013). Mutations of *OsWLP1* (Song et al. 2014) and *OsTCD11* (Wang et al. 2017b), which encode the 50S large subunit protein RPL13 and the 30S small subunit protein RPS6, respectively, causes impaired chloroplast development and an albino seedling phenotype at low temperature. Mutations of *OsASL2* (Lin et al. 2015b) and *OsAL1* (Zhao et al. 2016), which encode the 50S large subunit protein RPL21 and RPL12, respectively, cause impaired chloroplast development and seedling-lethal albino phenotype. Mutation of *OsWGL2*, which encodes the 30S small subunit protein RPS9, causes impaired chloroplast development and an albino seedling phenotype (mutants generated by CRISPR/Cas9) (Qiu et al. 2018).

Loss-of-function of *OsSLC1* especially precluded the intron splicing of *rps16* in the *slc1* mutant (Fig. 6a, b), which indicates that the post-transcriptional processing and translation of *rps16* was blocked in the mutant. Given the deficiency in Rps16 protein, the 30S small subunit is unable to recruit 16S rRNAs, which thus could be degraded, and result in severe reduction in the transcript levels of 16S rRNAs in the *slc1* mutant (Fig. 7a). Therefore, we infer that chloroplasts in the *slc1* mutant failed to assemble the normal 70S ribosomes, which resulted in severe defects in protein synthesis. As a result of the impairment in protein synthesis, the transcript levels of PEP genes, such as *rpoA*, *rpoB*, *rpoC1* and *rpoC2*, and those of plastid-encoded ribosome genes, such as *rpl2*, *rpl16*, *rpl23*, *rps2*, *rps12* and *rps15*, accumulated significantly in the *slc1* mutant (Fig. 7b). In addition, given the impediment in protein synthesis, chloroplasts in the *slc1* mutant possessed abnormal thylakoids and few grana stacks (Fig. 2d, f), which resulted in few photosynthetic pigments bound by abnormal thylakoids in the *slc1* mutant (Fig. 1b). Thus, photosynthesis was severely impaired in the *slc1* mutant (Fig. 2). In addition, the transcript levels of the photosynthesis-related genes *PORA* and *Cab2R* were significantly reduced in the *slc1* mutant (Fig. 7c). Taken together, the present results reveal that *OsSLC1* is indispensable for early chloroplast development and normal seedling survival in rice.

### Multiple Factors May Be Involved in the Intron Splicing of *rps16*

In the previous studies, several intron splicing factors have been identified to be involved in the intron splicing of *rps16*, such as ZmCRS2, ZmCAF1, ZmWTF1 and OsCFM3 (Asakura et al. 2008; Jenkins et al. 1997; Kroeger et al. 2009; Ostheimer et al. 2003). Such factors are also found in large ribonucleoprotein complexes containing other factors. In maize, CRS2 was found in a large ribonucleoprotein complex containing CAF1 (Ostheimer et al. 2003), and WTF1 in a large ribonucleoprotein complex containing both CAF1 and CFM3 (Kroeger et al. 2009). Thus, we infer that OsSLC1, OsCAF1, OsCFM3, OsCRS2 and OsWTF1 together with other factors may form a ribonucleoprotein complex to perform the intron splicing of *rps16* in rice chloroplast. However, further work is needed to test this hypothesis.

## Conclusion

We described a novel chloroplast-localized P subgroup PPR protein, OsSLC1, which harbors 12 PPR motifs. OsSLC1 functions in the intron splicing of multiple chloroplast group II introns, especially the *rps16* intron, and plays a vital role in early chloroplast development and seedling survival in rice.

## Methods

### Plant Materials and Growth Conditions

The *slc1* mutant was obtained from tissue-culture-derived plants of rice *Oryza sativa subsp. japonica* ‘Zhonghua 11’. The heterozygous *slc1* plants were preserved to reproduce the *slc1* mutants. All rice plants used in this study were grown in fields at Chongqing or in growth chambers under 12 h light (10,000 lx) at 30 °C and 12 h dark at 26 °C.

### Photosynthetic Pigment and Photosynthetic Parameter Measurements

Leaf photosynthetic pigment contents of the wild type and *slc1* mutant at the third-leaf stage were measured as previously described (Lichtenthaler 1987; Wang et al. 2016b). Fresh leaves (approximately 0.1 g fresh weight) at the third-leaf stage were cut into small pieces and soaked in 25 mL of 95% ethanol for 24 h at room temperature in the dark. Pigment contents were measured with a UV-1800PC (Mapada) spectrophotometer at 663, 645 and 470 nm. Three biological replicates were performed. Microsoft Office Excel 2016 was used to analyze data, and Student’s two-tailed paired t-test was used to determine the statistical significance of data between the wild type and *slc1* mutant, the same as below. Leaf photosynthetic parameters, consisting of net photosynthetic rate, stomatal conductance, intercellular CO_2_ concentration and transpiration rate, were measured at the third-leaf stage from 09:00 to 11:00 with an LI-6400 portable photosynthesis system (LI-COR, Lincoln, NE, USA) in accordance with the manufacturer’s instructions. Three biological replicates were performed.

### Transmission Electron Microscopy

The chloroplast ultrastructure of the wild type and *slc1* mutant leaves at the third-leaf stage was examined as previously described (Liu et al. 2007). Leaf samples were fixed with 2.5% glutaraldehyde and 1% OsO_4_, dehydrated in an ethanol series, and finally embedded in Spurr resin. The fixed and embedded samples were stained with uranyl acetate and alkaline lead citrate and then observed with a H-7500 transmission electron microscope (Hitachi, Tokyo, Japan).

### Map-Based Cloning of OsSLC1

The heterozygous *slc1* plants were crossed with *Oryza sativa subsp. indica* ‘Jinhui 1’ for genetic analysis. The F_1_ seeds were harvested from each individual plant, and mutants segregating in the F_2_ population were selected for gene mapping. Sequence polymorphisms between *Oryza sativa subsp. japonica* ‘Nipponbare’ and subsp. *indica* ‘93–11’ were used to develop insertion/deletion and simple sequence repeat molecular markers (Shen et al. 2004). Gene annotation and primer design for DNA and cDNA sequencing were performed on the basis of information obtained from the National Center for Biotechnology Information (NCBI, https://www.ncbi.nlm.nih.gov/) database and Gramene (http://gramene.org/) databases. Multiple sequence alignment was performed with Vector NTI Advance 10 (Invitrogen, USA; http://www.invitrogen.com/). All primers used in this study are listed in Supplementary Table S2.

### Vector Construction for the CRISPR/Cas9 System and Genetic Complementation

A modified tRNA-processing strategy based on the CRISPR/Cas9 system was used for knockout of *OsSLC1* in the wild type. The procedures were followed as previously described (Wang et al. 2018). Two gRNA target sites (CCGTGGGAGTCCTACGACCGCGG, CGGTGTCAAGCCGAATACCCCGG) were designed to construct the *OsSLC1*-Cas9 vector, which was transformed into the wild-type (‘Zhonghua 11’) calli using an *Agrobacterium*-mediated method (Hiei and Komari 2008).

For complementation of the *slc1* mutant, the full-length coding sequences of *OsSLC1* and EGFP were cloned into the pCAMRubi2 vector digested with *Eco*RI and *Hin*dIII to generate the OsSLC1-EGFP vector, which was driven by the *OsUbiquitin2* promoter (Wang et al. 2000; Wang et al. 2016b). The OsSLC1-EGFP vector was transformed into the *slc1* calli using an *Agrobacterium*-mediated method (Hiei and Komari 2008).

### Sequence and Phylogenetic Analysis

Subcellular location prediction was performed using TargetP (http://www.cbs.dtu.dk/services/TargetP/) and ChloroP (http://www.cbs.dtu.dk/services/ChloroP/). Protein structure prediction was performed using NCBI, PROSITE (http://prosite.expasy.org/prosite.html), and Smart (http://smart.embl-heidelberg.de/). Proteins homologous to OsSLC1 were obtained by BLAST and downloaded from NCBI and UniProt (http://www.uniprot.org/). A neighbor-joining tree was constructed with the software MEGA v7.0, and statistical support for the tree topology was assessed by means of a bootstrap analysis with 1000 replicates (Kumar et al. 2016).

### RNA Preparation and Quantitative Real-Time PCR Analysis

Total rice RNAs were extracted from roots, culms, sheaths, leaves, young panicles (white) and green panicles of the wild type plants at the heading stage using an RNAprep Pure Plant Kit (Tiangen Biotech). First-strand cDNA was synthesized from 1 μg total RNA using a PrimeScript RT Reagent Kit with gDNA Eraser (Perfect Real Time, TaKaRa). Quantitative real-time PCR was conducted using a TB Green Premix Ex Taq II (Tli RNaseH Plus) (TaKaRa) on a Bio-Rad CFX96 system according to the manufacturer’s instructions. The genes used for quantitative real-time PCR were consistent with the previous studies (Wang et al. 2016b; Zhang et al. 2017; Wang et al. 2018). *OsActin1* and *OsUbiquitin* were used as double internal controls, and relative transcript levels were analyzed using the 2^–ΔΔCT^ method (Livak and Schmittgen 2001). Three biological replicates were performed.

### Subcellular Localization of OsSLC1

The full-length coding sequence of *OsSLC1* was fused to the N-terminus of GFP in the pAN580 vector digested with *Spe*I and *Bam*HI to generate the pOsSLC1-GFP vector. The pOsSLC1-GFP vector and the empty vector pAN580 were transformed into rice protoplasts as previously described (Zhang et al. 2011). A confocal laser scanning microscope (LSM700, Zeiss, Jena, Germany) was used to observe GFP fluorescence.

### Chloroplast RNA Editing and RNA Splicing Analysis

Total RNA was extracted from the wild type and *slc1* mutant leaves at the third-leaf stage using the RNAprep Pure Plant Kit (Tiangen Biotech, Beijing, China), and treated with Recombinant DNase I (RNase-free) (TaKaRa, Tokyo, Japan). First-strand cDNA was synthesized with random hexamers using a PrimeScript II 1st Strand cDNA Synthesis Kit (TaKaRa) (for RT-PCR analysis), and synthesized with RT primer mix using a PrimeScript RT Reagent Kit with gDNA Eraser (Perfect Real Time, TaKaRa) (for quantitative real-time PCR analysis). cDNA was diluted to 50 ng·μL^− 1^ and used for PCR amplification with PrimeSTAR Max DNA Polymerase (TaKaRa). A total of 18 intron splicing analysis and 26 C-to-U RNA editing analysis were performed as previously described (Corneille et al. 2000; Inada et al. 2004; Tan et al. 2014; Zhang et al. 2017; Wang et al. 2018). The RT-PCR primers used for intron splicing and RNA editing analysis were consistent with the previous study (Wang et al. 2018). The quantitative real-time PCR primers used for intron splicing and unsplicing analysis were shown in the Supplementary Table S2. Each pair of primers were designed to cross each intron for splicing analysis, whereas at least one primer was designed to locate each intron for unsplicing analysis. Quantitative real-time PCR was performed as mentioned above. *psbA* was used as internal control, and relative transcript levels were analyzed using the 2^–ΔΔCT^ method (Livak and Schmittgen 2001). Three biological replicates were performed. The intron splicing efficiencies were analyzed using the log_2_ ratios of spliced to unspliced transcripts in the *slc1* mutant compared to the corresponding values in the wild type.

## Supplementary information

**Additional file 1 : Figure S1**. Quantitative real-time PCR analysis of chloroplast spliced genes in the wild type and *slc1* mutant leaves at the third-leaf stage. **Table S1**. Analysis of chloroplast RNA editing (C to U) in the wild type and *slc1* mutant. **Table S2**. Primer sequences used in this study.

## Abbreviations

PPR: pentatricopeptide repeat
CRISPR/Cas9: clustered regularly interspaced short palindromic repeats and CRISPR-associated protein 9
PEP: plastid-encoded RNA polymerase
