# Supplementary material for: OsSLC1 Encodes a Pentatricopeptide Repeat Protein Essential for Early Chloroplast Development and Seedling Survival
Source: Rice (N Y). 2020 Apr 15;13:25. doi: 10.1186/s12284-020-00385-5 (PMC7160225; doi:10.1186/s12284-020-00385-5)
Supplement: Supplementary file 1 — Additional file 1 : Figure S1. Quantitative real-time PCR analysis of chloroplast spliced genes in the wild type and slc1 mutant leaves at the third-leaf stage. Table S1. Analysis of chloroplast RNA editing (C to U) in the wild type and slc1 mutant. Table S2. Primer sequences used in this study. [file 12284_2020_385_MOESM1_ESM.docx]

Supplementary data for

*OsSLC1* encodes a pentatricopeptide repeat protein essential for early chloroplast development and seedling survival

Jun Lv^1, †^, Lianguang Shang^2, †^, Yun Chen^3, †^, Yao Han^4^, Xiaoyan Yang^4^, Shuzhang Xie^4^, Wenqin Bai^4^, Mingyu Hu^4^, Hong Wu^4^, Kairong Lei^4^, Ya’nan Yang^2^, Shengzhen Ge^1^, HaiPhuong Trinh^1^, Yi Zhang^3, 🖂^, Longbiao Guo^5, 🖂^, Zhongwei Wang^4, 5, 🖂^

^1^ College of Agronomy and Biotechnology, Southwest University, Chongqing 400715, China

^2^ Shenzhen Branch, Guangdong Laboratory for Lingnan Modern Agriculture, Genome Analysis Laboratory of the Ministry of Agriculture, Agricultural Genomics Institute at Shenzhen, Chinese Academy of Agricultural Sciences, Shenzhen 518124, China

^3^ State Key Laboratory for Conservation and Utilization of Bio-resources in Yunnan, Research Center for Perennial Rice Engineering and Technology in Yunnan, School of Agriculture, Yunnan University, Kunming 650500, China

^4^ Chongqing Key Laboratory of Adversity Agriculture Research, Biotechnology Research Center, Chongqing Academy of Agricultural Sciences, Chongqing 401329, China

^5^ State Key Laboratory of Rice Biology, China National Rice Research Institute, Zhejiang 310006, China


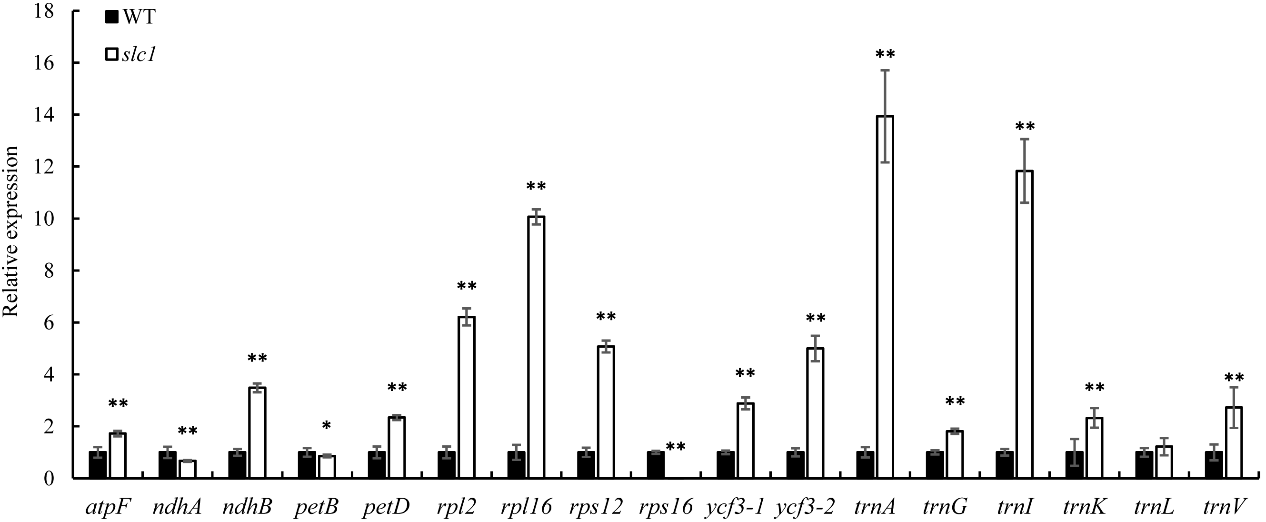


Figure S1 Quantitative real-time PCR analysis of chloroplast spliced genes in the wild type and *slc1* mutant leaves at the third-leaf stage. *psbA* was used as internal control, and relative transcript levels were analyzed using the 2^-ΔΔCT^ method. Three biological replicates were performed. Error bars are SDs. The asterisks indicate statistical significance between the wild type and *slc1* mutant, as determined by Student’s t-test (* *P* < 0.05, ** *P* < 0.01).

Table S1 Analysis of chloroplast RNA editing (C to U) in the wild type and slc1 mutant

| Gene | Editing position | Edited codon | Amino acid change | WT^a^ | *slc1*^a^ |
| --- | --- | --- | --- | --- | --- |
| *atpA* | C1148 | uCa | S→L | + | + |
| *matK* | C1252 | Cau | H→Y | + * | + * |
| *ndhA* | C473 | uCa | S→L | + | + * |
|  | C563 | uCa | S→L | + | + |
|  | C1070 | uCc | S→F | + * | + * |
| *ndhB* | C467 | cCa | P→L | + | + |
|  | C586 | Cau | H→Y | + | + |
|  | C611 | uCa | S→L | + | + |
|  | C704 | uCc | S→F | + | + |
|  | C737 | cCa | P→L | + * | + * |
|  | C830 | uCa | S→L | + | + |
|  | C836 | uCa | S→L | + | + |
|  | C1481 | cCa | P→L | + * | + |
| *ndhD* | C878 | uCa | S→L | + | + |
| *ndhF* | C62 | uCa | S→L | + | + |
| *ndhG* | C-10 | 5’-UTR | C→U | + | + |
|  | C347 | cCa | P→L | + | + * |
| *rpl2* | C2 | aCg | T→M | + * | + * |
| *rpoB* | C467 | uCg | S→L | + * | + * |
|  | C545 | uCa | S→L | + * | + * |
|  | C560 | uCa | S→L | + * | + * |
| *rpoC2* | C4106 | uCa | S→L | + | + * |
| *rps2* | C134 | aCa | T→I | + | + |
| *rps8* | C182 | uCa | S→L | + | + |
| *rps14* | C80 | uCa | S→L | + | + |
| *ycf3* | C185 | aCg | T→M | + | + |

^a^ Abbreviations: +, editing; -, no editing; *, partial editing.

Table S2 Primer sequences used in this study.

| Primers for mapping | | |
| --- | --- | --- |
| Primers | Forward sequence (5’-3’) | Reverse sequence (5’-3’) |
| R5814 | GCTCATCACTCCATGAATCG | ATCAAGGCTCGCTACTGCTC |
| R345 | ATTGGTAGCTCAATGCAAGC | GTGCAACAACCCCACATG |
| R3634-10 | TGAAGGCAGCACAGCAAA | AGGCCCAAACAATGGAAA |
| R4329-4 | TGGACGACAGGTGCCTAC | TCAAACGATACCGACGACT |
| Primers for sequencing | | |
| Primers | Forward sequence (5’-3’) | Reverse sequence (5’-3’) |
| SOsSLC1 | GATGTCAGGATCTCTCCGGGTTTCC | CACACGACAATGCCACCGAATCA |
| Primers for Cas9 vector construction | | |
| Primers | Sequences (5’-3’) | |
| U3tF | GTTACTAGATCGGGCCCAGGAATCTTTAAACATACGAACAGATCACT | |
| tRNAR1 | CGGTCGTAGGACTCCCACGGTGCACCAGCCGGGAATCGAAC | |
| gRNAF1 | CCGTGGGAGTCCTACGACCGGTTTTAGAGCTAGAAATAGCAAGTTAAAATAAG | |
| gRNAR1 | GCACCGACTCGGTGCCACTTTTTC | |
| tRNAF2 | GTGGCACCGAGTCGGTGCAACAAAGCACCAGTGGTCTAGTGGT | |
| tRNAR2 | GGGTATTCGGCTTGACACCGTGCACCAGCCGGGAATCGAAC | |
| gRNAF2 | CGGTGTCAAGCCGAATACCCGTTTTAGAGCTAGAAATAGCAAGTTAAAATAAG | |
| gRNAR2 | AGCTTGCATGCCTGCAGGGTAAAACGGAGGAAAATTCCATC | |
| Primers for subcellular vector construction | | |
| Primers | Forward sequence (5’-3’) | Reverse sequence (5’-3’) |
| OsSLC1-sub | GACAGCCCAGATCAACTAGTATGGATGCATACTACCTCGTCCG | CCCTTGCTCACCATGGATCCCAAATGTAACATTCCCCTTTGTTCC |
| Primers for complementary vector construction | | |
| Primers | Forward sequence (5’-3’) | Reverse sequence (5’-3’) |
| OsSLC1-com | ATTATTTCTTGCAGGATGGATGCATACTACCTCGTCCG | CGCCCTTGCTCACCATCAAATGTAACATTCCCCTTTGTTCC |
| EGFP-com | ATGGTGAGCAAGGGCGAGGAG | TCGATCGACAGATCATTACTTGTACAGCTCGTCCATGCC |
| Quantitative real-time PCR primers used for intron splicing and unsplicing analysis | | |
| Primers | Forward sequence (5’-3’) | Reverse sequence (5’-3’) |
| psbAF | GCGGTTCCCTATTCAGTGCTATG | TAACCATGAGCGGCCACAATATT |
| atpF-S ^a^ | GCTGGGAGTTTCGGGCTTAA | TTCGAGCTGCTCAATGGTTCC |
| atpF-U ^b^ | TGAGAGCCGAATGAGTCGAAAGA | TGCTCAATGGTTCCTCTACGCA |
| ndhA-S | ACGAGCTGCCGCTCAATCTAT | TAGGCTGACGCCAAAGATTCCA |
| ndhA-U | GGTTTTGGAATAGCGGTGGGA | GCTGACGCCAAAGATTCCATC |
| ndhB-S | CCTAGCCCCTTTTCATCAATGG | ATATCGAGAATTCGCGTGGCT |
| ndhB-U | GCCGTGCGAGATGAAAGTCTC | ATATCGAGAATTCGCGTGGCT |
| petB-S | GGTTTCGGTATCTCTGGAATATGAGT | AACAAGTAAGTGTAATTCCCCCTAAACA |
| petB-U | AATTCTCATATACGGTTCTCGGAGG | AACAAGTAAGTGTAATTCCCCCTAAACA |
| petD-S | AAAAGAAGGCGGATTATGGGAGT | ACGCGGGTTCCCCGTAATAA |
| petD-U | CTCATGTCCGGTTCCTTTGGG | ACGCGGGTTCCCCGTAATAA |
| rpl2-S | ATATCAATGGGAAATGCCCTACCT | CCTGCTGCTCTAGCTAATTGCC |
| rpl2-U | ACATCTAGAAAGCCGTATGCTTTGG | CCACCCCTTCCACGTGTGATT |
| rpl16-S | ATTGCTATGCTTAGTCCCAAAAGAAC | CTATCTGCCTCGCCGTAATCC |
| rpl16-U | CATTTGATTCGCGAGGAGCTG | GCCTCGGTAGGATTTTCCCTT |
| rps12-S | AGGGTTAAGGATTTACCCGGTG | GGCTTTTTGACCCCATATTTAGAAC |
| rps12-U | AATCGTCAACAAGGGCGTTCTA | CTGCTCCAGTCCCCTTACGAA |
| rps16-S | ATGTGGTAGAAAGCAACAAGCTGT | AGGTTGAGCACCTTTTTCAAGGA |
| rps16-U | TTCTCGAGCCGTATGAGGAGAA | TGCAACGATTCGATAGACAGCT |
| ycf3-1-S | TGCGAATAATTCCGACAACCTCC | TTGTGTGTATAAGGCCTATGTTATAGAGTA |
| ycf3-1-U | ACAGAAGGGAGGAGCCGTATGA | ATGCTCTCCATTGCTTGTGTGTAT |
| ycf3-2-S | ATTTCCGGGCACTAGAACGAA | TGTAGAATGGCCTGTTCTCCGT |
| ycf3-2-U | TTTCTGAGCCGTATGAGGTAGGAA | TCACCCTGTAGAATGGCCTGTTC |
| trnA-S | GGGGATATAGCTCAGTTGGTAGAG | TGGAGATAAGCGGACTCGAAC |
| trnA-U | ATCGTTGGCCTCTATGGTAGAAC | TGGAGATAAGCGGACTCGAAC |
| trnG-S | GCGGGTATAGTTTAGTGGTAAAAGCC | GCATCGTTAGCTTGGAAGGCTAG |
| trnG-U | GTGGTGAGAATACCCATAAAGGAG | GGGTAGCGGGAATCGAACC |
| trnI-S | GGGCTATTAGCTCAGTGGTAGAG | TGGGCCATCCTGGACTTGA |
| trnI-U | AGAGCGCAGTACAACGGAGAG | TGGGCCATCCTGGACTTGA |
| trnK-S | CTGGGTTGCTAACTCAATGGTAGAG | GTTGCCCGGGACTCGAAC |
| trnK-U | TTTCTACATAGGGAAAGTCGTGTGC | AGTTTCCATATGGGTTGCCCG |
| trnL-S | GGGATATGGCGAAATCGGTAGAC | TGGGGATAGAGGGACTTGAACC |
| trnL-U | AATCGGACGAGGATAAAGAGAGAG | TGGGGATAGAGGGACTTGAACC |
| trnV-S | AGGGCTATAGCTCAGTTCGGTAG | TAGGGCTATACGGATTCGAACC |
| trnV-U | TCTATCAGTTGATAAAAGAGCCCAA | TAGGGCTATACGGATTCGAACC |
| Note: The underlined bases are overlap sequences designed as per the *pEASY*-Uni Seamless Cloning and Assembly Kit user manual. ^a^ S, splicing; ^b^ U, unsplicing | | |
